# Proliferative glomerulonephritis with monoclonal immunoglobulin G3 lambda deposits accompanied by glomerular positive staining for nephritis-associated plasmin receptor and related plasmin activity: A report of two cases

**DOI:** 10.3389/fmed.2022.1059575

**Published:** 2023-01-12

**Authors:** Takahiro Uchida, Takashi Oda, Takahiko Hoshino, Takashi Sakai, Aki Kojima, Dan Inoue, Tadasu Kojima, Muneharu Yamada

**Affiliations:** Department of Nephrology and Blood Purification, Kidney Disease Center, Tokyo Medical University Hachioji Medical Center, Hachioji, Tokyo, Japan

**Keywords:** infection-related glomerulonephritis, nephritis-associated plasmin receptor, nephrotic syndrome, plasmin, proliferative glomerulonephritis with monoclonal immunoglobulin G deposits

## Abstract

Proliferative glomerulonephritis with monoclonal immunoglobulin (Ig) G deposits (PGNMID) is a relatively uncommon entity of monoclonal gammopathy of renal significance, and its detailed pathogenesis is not well understood. We, herein, report two cases of patients with PGNMID; their renal biopsy showed glomerular histological features of membranoproliferative glomerulonephritis pattern with endocapillary proliferation accompanied by non-organized granular electron-dense deposits that consisted of monoclonal IgG3-lambda. Neither symptomatic episodes of preceding infection nor infection foci were found in both patients; however, glomerular positive staining for nephritis-associated plasmin receptor (NAPlr) and related plasmin activity were observed. Although NAPlr was originally considered as a candidate nephritogenic protein for post-streptococcal acute glomerulonephritis, its positive staining and related plasmin activity have been observed in glomeruli of various cases with bacterial infection-related glomerulonephritis and is considered to be a general histological biomarker of infection-related glomerulonephritis. The present cases suggest that evaluation of immunoreactivity for NAPlr and related plasmin activity in glomeruli provides an important clue regarding the infection-related pathogenesis of PGNMID.

## Introduction

Proliferative glomerulonephritis with monoclonal immunoglobulin (Ig) G deposits (PGNMID) is a novel form of glomerulonephritis that is accompanied by monoclonal IgG deposition (1, 2). Although PGNMID was originally considered to be IgG3-driven, it was thereafter suggested that this disease is not restricted to patients with monoclonal IgG deposition, because IgA (3), IgM (4), and light chain-driven cases (5) have also been reported. Its usual histological features are membranoproliferative glomerulonephritis (MPGN) and endocapillary proliferative glomerulonephritis.

Since the term monoclonal gammopathy of renal significance (MGRS) was proposed in 2012 to distinguish monoclonal gammopathy resulting in the development of renal disease from that without renal involvement (6), various renal diseases have been described as associated with MGRS, and PGNMID is now categorized into a type of MGRS (7). Nevertheless, the detection rate of monoclonal gammopathy in patients with PGNMID is relatively low (reportedly only 30% of the cases) (2), and the true nature of its pathogenesis and treatment has not been elucidated. The renal prognosis is generally poor, whereas it has also been reported that some cases show favorable renal outcomes by conservative therapy alone or following the management of infections (1, 2, 8).

Herein, we report two cases of IgG3-lambda type PGNMID in which positive staining for nephritis-associated plasmin receptor (NAPlr) and related plasmin activity, recently proposed diagnostic biomarkers of bacterial infection-related glomerulonephritis (IRGN) (9), were observed in the glomeruli.

## Case presentation

### Case 1

A 72-year-old Japanese man without history of renal disease experienced an 8-kg increase in body weight and developed edema in his legs. Massive proteinuria was detected at a clinic, and he was referred to our hospital.

The initial laboratory results are summarized in Table 1. Urinalysis showed a protein level of 12.2 g/day, and hypoproteinemia and hypogammaglobulinemia were noted; however, monoclonal protein was not detected. Hypocomplementemia was absent, and the titers for antinuclear antibody, antistreptolysin O (ASO), and anti-parvovirus B19 (PVB19) IgM antibody were within the normal range. Computed tomography did not show evidence of bone or lymphoproliferative lesions or foci of infection.

**Table 1:** Laboratory data of the patients.

		**CASE 1**	**CASE 2**
Urinalysis	Hematuria	30–49/HPF	10–19/HPF
	Proteinuria	12.22 g/day	3.4 g/day
Complete blood count	White blood cell (4,000–8,000/μL)	8,660/μL	3,660/μL
	Hemoglobin (13.5–17.5 g/dL)	13.5 g/dL	11.9 g/dL
	Platelet (150,000–350,000/μL)	300,000/μL	154,000/μL
Biochemistry	Creatinine (0.65–1.07 mg/dL)	1.30 mg/dL	1.13 mg/dL
	Blood urea nitrogen (8–20 mg/dL)	14.2 mg/dL	13.0 mg/dL
	Total protein (6.6–8.1 g/dL)	4.5 g/dL	4.4 g/dL
	Albumin (4.1–5.1 g/dL)	2.3 g/dL	2.2 g/dL
Serology	IgG (861–1,747 mg/dL)	215 mg/dL	486 mg/dL
	IgA (93–393 mg/dL)	93 mg/dL	128 mg/dL
	IgM (33–183 mg/dL)	34 mg/dL	21 mg/dL
	SPEP/UPEP	N.D./N.D.	IgG-lambda/IgG-lambda
	Serum FLC ratio (0.26–1.65)	1.68	1.31
	Complement C3 (73–138 mg/dL)	81.9 mg/dL	52.7 mg/dL
	Complement C4 (11–31 mg/dL)	19.5 mg/dL	16.8 mg/dL
	ANA (<40)	<40	40
	dsDNA-IgG (<12 IU/mL)	<10 IU/mL	<10 IU/mL
	MPO-ANCA (<3.5 U/mL)	<1.0 U/mL	<1.0 U/mL
	PR3-ANCA (<3.5 U/mL)	<1.0 U/mL	<1.0 U/mL
	C-reactive protein (≤0.14 mg/dL)	<0.02 mg/dL	<0.02 mg/dL
	ASO (<239 IU/mL)	<10 IU/ML	36 IU/mL
	PVB19 IgM (<0.8)	0.23	0.20

ANA, antinuclear antibody; ANCA, anti-neutrophil cytoplasmic antibody; ASO, Antistreptolysin O; dsDNA, double-stranded DNA; FLC, free light-chain; HPF, high-power field; Ig, immunoglobulin; MPO, myeloperoxidase; N.D., not detected; PR3, proteinase3; PVB19, parvovirus B19; SPEP, serum protein electrophoresis; UPEP, urine protein electrophoresis.

**Figure 1:**
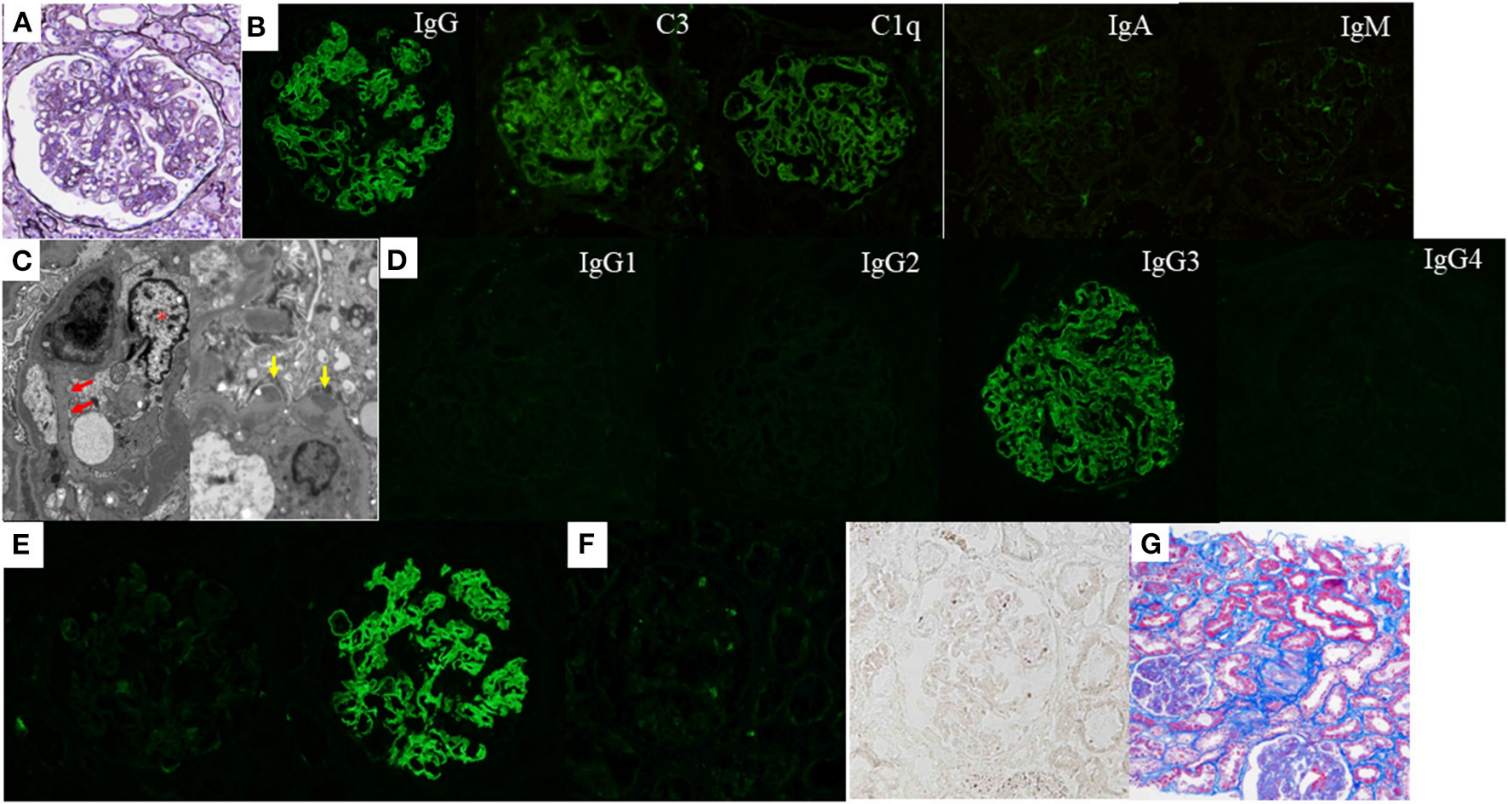
Histological features of the renal biopsy of case 1. **(A)** Light microscopy section shows a glomerulus with mesangial expansion and endocapillary proliferation as well as thickening and double contour of capillary walls (periodic acid-methenamine-silver stain). **(B)** Strong deposition of immunoglobulin (Ig) G, complement C3, and C1q are observed along the glomerular capillary walls by immunofluorescence (IF) staining. The deposition of IgA and IgM was ambiguous. **(C)** Subendothelial and mesangial electron-dense deposits are shown by electron microscopy. Newly formed glomerular basement membrane (red arrows) and infiltration of macrophages (asterisk) are also observed (left). Small amounts of subepithelial electron-dense deposits (yellow arrows) were also observed (right). **(D)** IF staining for IgG subclasses demonstrates sole deposition of IgG3. **(E)** There is no deposition of kappa-light chain by IF staining (left), whereas strong deposition of lambda-light chain is observed (right). **(F)** Both IF staining for nephritis-associated plasmin receptor (left) and *in situ* zymography for plasmin activity (right) are positively observed with a similar distribution in a glomerulus. **(G)** The degree of interstitial fibrosis and tubular atrophy are mild (Masson's trichrome stain).

A percutaneous renal biopsy was performed, and light microscopy (LM) showed sections containing 26 glomeruli. One glomerulus showed global sclerosis, while the rest showed diffuse and global MPGN patterns with mesangial expansion, endocapillary proliferation, and thickening and double contour of capillary walls (Figure 1A). Immunofluorescence (IF) staining showed deposition of IgG, complement C3, and C1q mainly along the glomerular capillary walls, whereas the deposition of IgA and IgM was ambiguous (Figure 1B). Non-organized electron-dense deposits (EDD) were found mainly in the subendothelial and mesangial areas on electron microscopy (EM), but small amounts of subepithelial EDD were also observed (Figure 1C). IgG3 deposition was solely observed on IF IgG subclass staining (Figure 1D), and IF staining for light chains demonstrated the deposition of only lambda chains without kappa chains (Figure 1E). Based on these findings, the patient was diagnosed with IgG3-lambda type PGNMID. Furthermore, glomerular positive staining for NAPlr and plasmin activity were observed on IF staining and *in situ* zymography, respectively (Figure 1F). The degree of interstitial fibrosis and tubular atrophy was mild (Figure 1G).

Although involvement of bacterial infection in the pathogenesis of his renal disease was suggested, preceding infection was absent, and signs of infection were not detected through repeated examinations. Steroid therapy was started, which substantially improved his nephrotic syndrome. However, it was exacerbated immediately after the cessation of steroids, whereas hypocomplementemia had never been observed. Steroid treatment was restarted, and he showed clinical improvement, which was maintained during the 1-year follow-up period (serum creatinine level, 1–1.5 mg/dL).

### Case 2

A 59-year-old Japanese woman with treatment history of hypertension for 1 year suddenly developed peripheral edema. Primary care doctor detected hematuria and proteinuria, and she was referred to our hospital.

The initial laboratory results are summarized in Table 1. Massive proteinuria and hypoproteinemia were noted. Serum and urine protein electrophoresis revealed a monoclonal IgG-lambda peak, but bone marrow aspiration showed <10% plasma cells, which did not meet the diagnostic criteria of multiple myeloma. Although C3 hypocomplementemia was present, titers for antinuclear antibody, anti-double-stranded DNA antibody, ASO, and anti-PVB19 IgM antibody were within the normal range.

**Figure 2:**
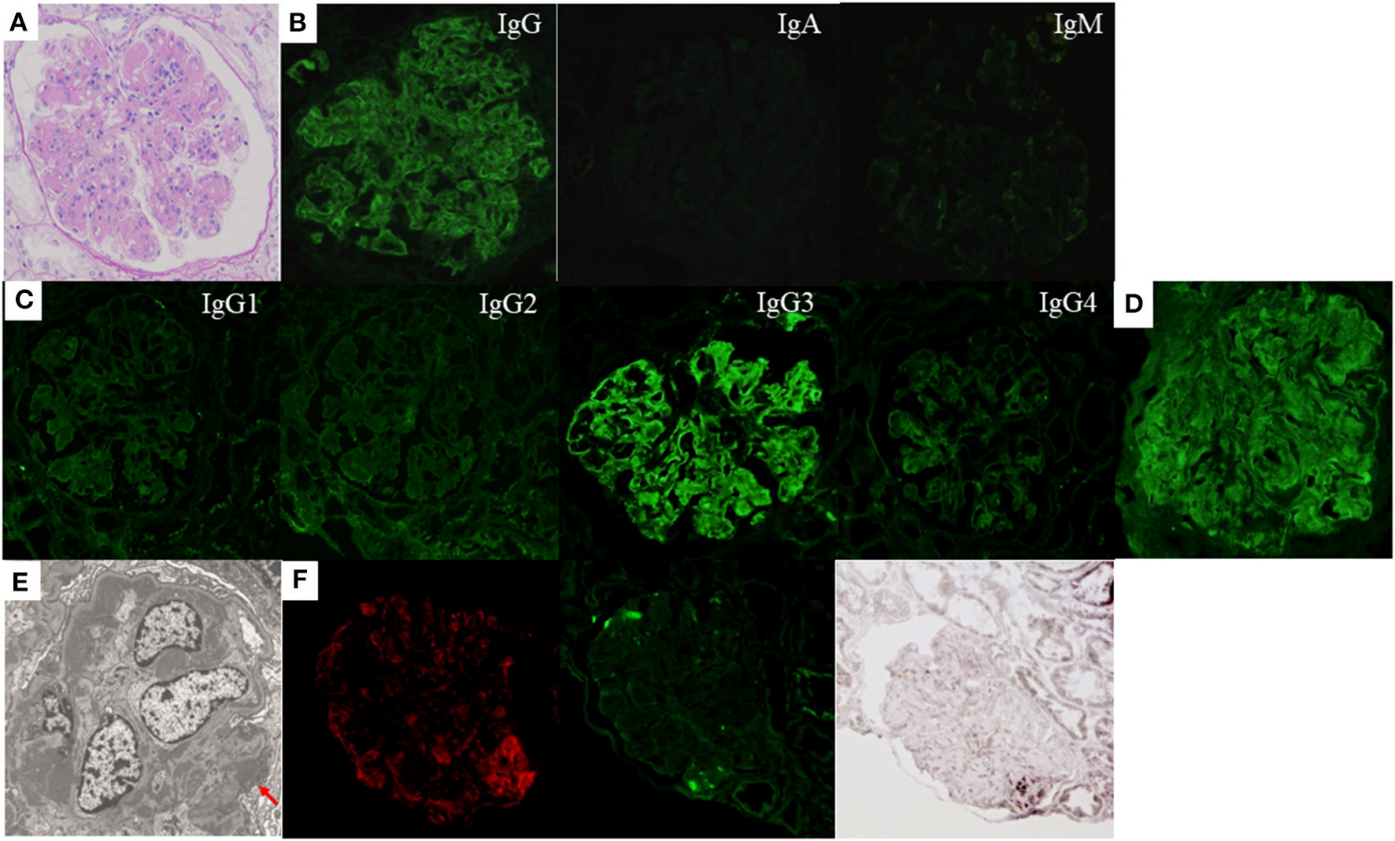
Histological features of the first renal biopsy of case 2. Lobulation of a glomerulus accompanied by mesangial and endocapillary proliferation as well as thickening and double contour of capillary walls are shown by light microscopy [**(A)** periodic acid-Schiff stain]. Immunofluorescence (IF) staining showed strong deposition of immunoglobulin (Ig) G mainly along the glomerular capillary walls. The deposition of IgA and IgM was negative **(B)**. IF staining for IgG subclasses demonstrates sole deposition of IgG3 **(C)**. Strong deposition of lambda-light chain is observed by IF staining **(D)**, which seems to correspond with electron-dense deposits in the mesangial and subendothelial areas shown by electron microscopy. There were also small amounts of subepithelial electron-dense deposits [**(E)** red arrow]. Positive IF staining for complement C3 (left), nephritis-associated plasmin receptor (NAPlr, middle), and *in situ* zymography for plasmin activity (right) are shown in the glomerulus. The distribution of positive portion for C3 and NAPlr is different, but that for NAPlr and plasmin activity is similar **(F)**.

The first renal biopsy showed MPGN pattern with endocapillary proliferation (Figure 2A). Only one glomerulus was obsolescent in LM sections containing 20 glomeruli. Congo red staining was negative. IF staining for IgG was positive mainly along the glomerular capillary walls, whereas that for IgA and IgM was both negative (Figure 2B). IF IgG subclass staining demonstrated sole deposition of IgG3 (Figure 2C). Strong lambda-light chain deposition on IF staining (Figure 2D) and corresponding EDD mainly in the mesangial and subendothelial areas were observed (Figure 2E). There were also small amounts of subepithelial EDD (Figure 2E). On the other hand, IF kappa-light chain staining was rather weak and seemed non-specific (data not shown). Positive staining for NAPlr and plasmin activity was observed in the glomeruli with a similar distribution, but the staining pattern of them was different from that of complement C3 (Figure 2F). Neither fibrils nor powdery deposits were observed under EM, and no EDD were found on TBM.

**Figure 3:**
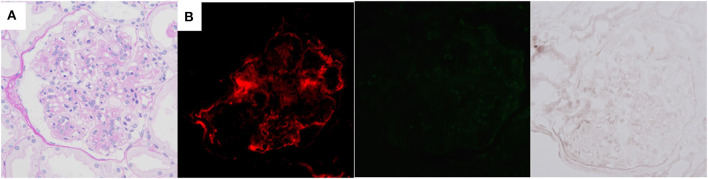
Histological features of the second renal biopsy of case 2, performed about 3 years after the first. Mesangial proliferation and focal double contour of capillary walls are shown by light microscopy [**(A)** periodic acid-Schiff stain]. Immunofluorescence staining for complement C3 is positive (left), but both nephritis-associated plasmin receptor (middle) and plasmin activity (right) are negative **(B)**.

Although infection-related PGNMID was suspected, detailed examination could not detect the focus of infection. Therefore, we started steroid treatment, and partial remission of nephrotic syndrome (proteinuria level of about 0.5 g/g Cr) was observed in association with normalization of serum C3 levels. The dose of steroids was gradually tapered and retained at 5 mg daily, which maintained her remission for 2 years. However, severe nephrotic syndrome relapsed (proteinuria level, 5–10 g/day) in association with decrease in the C3 level (63.7 mg/dL). The second renal biopsy was performed about 3 years after the first and showed similar pathological findings to those of the first (Figure 3A), but 5 out of 26 glomeruli were globally sclerotic in LM sections, suggesting the progression of histological chronic lesions. NAPlr and plasmin activity were completely negative at this time (Figure 3B). Although the dose of steroids was increased, the patient was complicated by severe hypogammaglobulinemia (IgG level, 100–300 mg/dL), which made it difficult for us to further intensify immunosuppressive therapy. The development of overt multiple myeloma was ruled out by the feature of repeat bone marrow aspiration that was performed about 4 years after the first. Her condition progressed to end-stage renal disease (ESRD), and she became dialysis-dependent 5 years after disease onset.

## Discussion

Renal histological features of the two patients, i.e., glomerulopathy of MPGN pattern on LM, IgG3-lambda sole deposition on IF staining, and subendothelial and mesangial EDD on EM, are typical characteristics of PGNMID. Furthermore, endocapillary proliferative change with positive glomerular staining for NAPlr and plasmin activity were shown in both patients, which we considered as a novel finding.

NAPlr was originally isolated from cytoplasmic fraction of group A streptococcus as a candidate nephritogenic protein for poststreptococcal acute glomerulonephritis (PSAGN) and was also found to be the same molecule as streptococcal glyceraldehyde-3-phosphate dehydrogenase (GAPDH) (10). NAPlr accumulates on the inner side of the glomerular capillary walls and traps plasmin and maintains its activity in the glomeruli, thereby causing glomerular damage by degrading extracellular matrix proteins and by activating and accumulating inflammatory cells, such as neutrophils and macrophages (11). NAPlr and related plasmin activity are assumed to be involved in the development of glomerular lesions, especially at the initial phase. Indeed, glomerular positive staining for NAPlr and plasmin activity was frequently observed in the early-phase PSAGN patients and was initially considered as specific histological markers for PSAGN, although these markers usually disappear at the later phase (12). They reportedly disappear within 30 days after the disease onset in patients with PSAGN, and therefore it could be possible that NAPlr and plasmin activity were not observed in glomerular lesions, if the renal biopsy was not performed during the acute phase.

Subsequent studies have revealed that similar glomerular staining patterns are also observed in patients with other types of glomerular diseases in association with streptococcal infections (e.g., C3 glomerulopathy, MPGN, antineutrophil cytoplasmic antibody-associated vasculitis, or IgA vasculitis) as well as non-streptococcal infections (9). It has been shown that sequence of GAPDH of such non-streptococcal bacteria have high similarity to that of streptococcal GAPDH (NAPlr) and that such GAPDH also possess plasmin-binding capacity (9). Thus, it is reasonable that the deposition of GAPDH from these bacteria in glomeruli is detected by anti-NAPlr antibody due to its cross-immunoreactivity, and positive plasmin activity would also be observed due to plasmin-binding capacity of the GAPDH. Therefore, glomerular positive staining for NAPlr and plasmin activity are now suggested as general diagnostic biomarkers of bacterial IRGN (9). Considering that the international diagnostic criteria for IRGN has not been established yet, although Nasr et al. proposed the tentative criteria (13), and the diagnosis of IRGN in the elderly is often difficult due to the lack of definitive symptoms and specific markers, NAPlr and plasmin activity are of high value in the diagnosis of IRGN. Indeed, various forms of glomerulonephritis with positive staining for NAPlr and plasmin activity have recently been reported, even if the causative pathogens of infection could not be identified (9, 14, 15). The specificity of anti-NAPlr antibody for bacterial GAPDH has also been confirmed by Western blotting (12). Furthermore, as far as we know, positive staining for NAPlr has been reported only in glomerular lesions with endocapillary proliferation, with distinct C3 deposition, and with EDD. Based on such observations, glomerular positive staining for NAPlr is considered to be specific for glomerular diseases associated with infection. However, whether glomerular positive staining for NAPlr and plasmin activity would also be observed in patients with non-bacterial IRGN, such as viral IRGN, or not remains to be solved in future studies. Although only small amounts of subepithelial EDD were observed in the renal biopsy tissues of our patients, it is not so rare for the limited renal biopsy tissues of IRGN patients not to detect typical humps, and actually we have previously reported a case of a patient with PSAGN in which renal biopsy showed atypical massive subendothelial deposits without subepithelial humps (16). The differences in responses of the affected hosts, including genetic and/or acquired background of the complement system, may affect the specific histology (17), especially in the later phase of disease course.

We did not evaluate whether bacterial components other than NAPlr were present or not in the renal biopsy tissues of our patients, which we considered as a limitation. However, in our patients, the levels of ASO were normal and it was suggested that some bacterial strains other than *Streptococci* with NAPlr-like GAPDH function were involved in the development of glomerulonephritis. We have previously reported that the staining intensity of NAPlr and plasmin activity in patients with non-streptococcal IRGN is generally weaker than that in patients with PSAGN (9). In this regard, the timing when renal biopsy is performed might affect. Because positive immunostaining of NAPlr in patients with non-streptococcal IRGN is presumably caused by the deposition of bacterial GAPDH which is detected by cross-immunoreactivity to the anti-NAPlr antibody, the staining intensity might become weak.

To date, there has been only one reported case of PGNMID in which glomerular positive staining for NAPlr and plasmin activity were observed (18). Renal function of the reported patient, who had complement factor H mutation that could attribute to the dysregulation of complement alternative pathway, recovered by conservative therapy, and it is suggested that infection-related PGNMID and IRGN share common clinical features and pathogenesis.

There have also been several reported cases of PGNMID that were associated with infection, including PVB19 (8, 19) or corona virus disease 2019 (20). More intriguingly, preceding or concurrent infection was identified in more than half of the pediatric patients with PGNMID (21). Thus, infection could be deeply involved in the pathogenesis of PGNMID, more than previously considered. In this regard, it should be kept in mind that the identification of infectious foci is not uncommonly difficult in adult patients.

Effective treatment strategy for PGNMID has not been established yet, and its renal prognosis is generally guarded (2). Older age, higher creatinine level, and higher histological chronicity are reportedly associated with poor prognosis (2).

Favorable renal outcome has been observed by steroid monotherapy at least on short-term follow-up in our aged patient with PGNMID who did not show severe renal dysfunction or histological chronicity (case 1). Controlling the underlying infection is most important for the treatment of IRGN, but immunosuppressive therapy could be a therapeutic option if the infection is under control (22). It is also noteworthy that treatment of infection did not bring clinical improvement of PGNMID in pediatric patients associated with infection (21). On the other hand, our middle-aged patient (case 2) progressed to ESRD despite steroid therapy. NAPlr/plasmin activity was positive in the first biopsy but was negative in the second biopsy, suggesting that the infection would be mainly involved in the initiation phase of PGNMID and that responses of the hosts, such as accumulating inflammatory cells, immune complexes, and complements (including both genetic and acquired background of the complement system) continue to play important roles at the later phase even after the disappearance of these markers. A clone-directed approach that is reported to improve renal outcome (23) may have been a better choice for the patient. In addition, the detailed investigation of alternative complement pathway, including complement factor H (CFH) level, anti-CFH antibody, or CFH-related proteins (1, 3) etc., was important in case 2, because both the onset and relapse of nephrotic syndrome were associated with C3 hypocomplementemia. However, we could not assess them because evaluation of these elements is not performed in usual clinical examination in Japan, which we considered as another limitation.

Infection-related findings were limited in our patients and steroid treatment did not disclose the underlying infections. In this regard, similar conditions actually occur in patients of renal diseases with focal infection, such as IgA nephropathy. IgA nephropathy is now considered as a disease of focal infection in palatine tonsils and therefore tonsillectomy is widely performed in Japan (24); however, patients with IgA nephropathy seldom present with symptoms related to tonsillitis, and immunosuppressive therapy is widely performed without overt manifestations of the tonsil infection. Another possibility is that our patients already got spontaneous remission of the infections when PGNMID was diagnosed, as with the case of most PSAGN patients.

In conclusion, we report two cases of PGNMID with glomerular positive staining for NAPlr and plasmin activity. Infections are considered to play an important role in the disease pathogenesis of PGNMID. Evaluating immunoreactivity for anti-NAPlr antibody and plasmin activity in glomeruli would provide an important clue for patients with PGNMID.

## Patients' perspectives

Case 1 has remained in a stable and improved condition, as his peripheral edema and other presenting symptoms resolved after steroid treatment. Case 2 who showed monoclonal IgG-lambda protein has been dialysis-dependent, but development of hematologic malignancy has not been observed.

## Data availability statement

The original contributions presented in the study are included in the article/supplementary material, further inquiries can be directed to the corresponding author.

## Ethics statement

Written informed consent was obtained from the patients for the publication of any potentially identifiable images or data included in this article.

## Author contributions

Writing the manuscript draft: TU. Manuscript revision: TO and MY. Clinical care of the patient: TH, TS, AK, and TK. Histological analysis: TU, DI, and TO. All authors contributed to the article and approved the submitted version.

## References

[1] Nasr SH Markowitz GS Stokes MB Seshan SV Valderrama E Appel GB. Proliferative glomerulonephritis with monoclonal IgG deposits: a distinct entity mimicking immune-complex glomerulonephritis. Kidney Int. (2004) 65:85–96. 10.1111/j.1523-1755.2004.00365.x 14675039

[2] Nasr SH Satoskar A Markowitz GS Valeri AM Appel GB Stokes MB. Proliferative glomerulonephritis with monoclonal IgG deposits. J Am Soc Nephrol. (2009) 20:2055–64. 10.1681/ASN.2009010110 19470674 PMC2736767

[3] Vignon M Cohen C Faguer S Noel LH Guilbeau C Rabant M. The clinicopathologic characteristics of kidney diseases related to monotypic IgA deposits. Kidney Int. (2017) 91:720–8. 10.1016/j.kint.2016.10.026 28069266

[4] Yahata M Nakaya I Takahashi S Sakuma T Sato H Soma J. Proliferative glomerulonephritis with monoclonal IgM deposits without Waldenstrom's macroglobulinemia: case report and review of the literature. Clin Nephrol. (2012) 77:254–60. 10.5414/CN107230 22377259

[5] Komatsuda A Nara M Ohtani H Nimura T Sawada K Wakui H. Proliferative glomerulonephritis with monoclonal immunoglobulin light chain deposits: a rare entity mimicking immune-complex glomerulonephritis. Intern Med. (2012) 51:3273–6. 10.2169/internalmedicine.51.8513 23207123

[6] Leung N Bridoux F Hutchison CA Nasr SH Cockwell P Fermand JP. Monoclonal gammopathy of renal significance: when MGUS is no longer undetermined or insignificant. Blood. (2012) 120:4292–5. 10.1182/blood-2012-07-445304 23047823

[7] Leung N Bridoux F Batuman V Chaidos A Cockwell P D'Agati VD. The evaluation of monoclonal gammopathy of renal significance: a consensus report of the international kidney and monoclonal gammopathy research group. Nat Rev Nephrol. (2019) 15:45–59. 10.1038/s41581-018-0077-4 30510265 PMC7136169

[8] Fujita E Shimizu A Kaneko T Masuda Y Ishihara C Mii A. Proliferative glomerulonephritis with monoclonal immunoglobulin G3kappa deposits in association with parvovirus B19 infection. Hum Pathol. (2012) 43:2326–33. 10.1016/j.humpath.2012.04.004 22819999

[9] Uchida T Oda T. Glomerular deposition of nephritis-associated plasmin receptor (NAPlr) and related plasmin activity: key diagnostic biomarkers of bacterial infection-related glomerulonephritis. Int J Mol Sci. (2020) 21:72595. 10.3390/ijms21072595 32276523 PMC7178002

[10] Yoshizawa N Yamakami K Fujino M Oda T Tamura K Matsumoto K. Nephritis-associated plasmin receptor and acute poststreptococcal glomerulonephritis: characterization of the antigen and associated immune response. J Am Soc Nephrol. (2004) 15:1785–93. 10.1097/01.ASN.0000130624.94920.6B 15213266

[11] Oda T Yamakami K Omasu F Suzuki S Miura S Sugisaki T. Glomerular plasmin-like activity in relation to nephritis-associated plasmin receptor in acute poststreptococcal glomerulonephritis. J Am Soc Nephrol. (2005) 16:247–54. 10.1681/ASN.2004040341 15574512

[12] Yoshizawa N Yamada M Fujino M Oda T. Nephritis-associated plasmin receptor (NAPlr): an essential inducer of C3-dominant glomerular injury and a potential key diagnostic biomarker of infection-related glomerulonephritis (IRGN). Int J Mol Sci. (2022) 23:9974. 10.3390/ijms23179974 36077377 PMC9456382

[13] Nasr SH Radhakrishnan J D'Agati VD. Bacterial infection-related glomerulonephritis in adults. Kidney Int. (2013) 83:792–803. 10.1038/ki.2012.407 23302723

[14] Komaki K Shiotsu Y Adachi H Urata N Hara M Nakayama M. Nephritis-associated plasmin receptor (NAPlr)-positive glomerulonephritis in a case of ANCA-negative small vessel vasculitis. CEN Case Rep. (2022) 11:90–6. 10.1007/s13730-021-00635-5 34389964 PMC8811102

[15] Han W Suzuki T Watanabe S Nakata M Ichikawa D Koike J. Galactose-deficient IgA1 and nephritis-associated plasmin receptors as markers for IgA-dominant infection-related glomerulonephritis: a case report. Medicine. (2021) 100:e24460. 10.1097/MD.0000000000024460 33592898 PMC7870202

[16] Uchida T Oda T Watanabe A Izumi T Higashi K Kushiyama T. Clinical and histologic resolution of poststreptococcal glomerulonephritis with large subendothelial deposits and kidney failure. Am J Kidney Dis. (2011) 58:113–7. 10.1053/j.ajkd.2011.04.011 21684437

[17] Oda T Yoshizawa N. Factors affecting the progression of infection-related glomerulonephritis to chronic kidney disease. Int J Mol Sci. (2021) 22:905. 10.3390/ijms22020905 33477598 PMC7831296

[18] Takehara E Mandai S Shikuma S Akita W Chiga M Mori T. Post-infectious proliferative glomerulonephritis with monoclonal immunoglobulin G deposits associated with complement factor H mutation. Intern Med. (2017) 56:811–7. 10.2169/internalmedicine.56.7778 28381748 PMC5457925

[19] Santana de Roberts R Batal I Aljareh A Jim B. Proliferative glomerulonephritis with monoclonal immunoglobulin deposits associated with parvovirus B19. BMJ Case Rep. (2021) 14:61. 10.1136/bcr-2021-243061 34158330 PMC8220517

[20] Shieh M Giannini JA Combs SA Shaffi SK Messias NC Teixeira JP. Proliferative glomerulonephritis with monoclonal immunoglobulin deposits triggered by COVID-19: a case report. CEN Case Rep. (2022) 2022:1–6. 10.1007/s13730-022-00687-1 35122206 PMC8815387

[21] Miller P Xiao AY Kung VL Sibley RK Higgins JP Kambham N. Progression of proliferative glomerulonephritis with monoclonal IgG deposits in pediatric patients. Pediatr Nephrol. (2021) 36:927–37. 10.1007/s00467-020-04763-5 33044675

[22] Okumura M Sugihara S Seki K Nagaoka K Okawa N Ebihara M. Use of immunosuppressive therapy in the treatment of IgA-dominant infection-related glomerulonephritis. Intern Med. (2022) 61:697–701. 10.2169/internalmedicine.7404-21 35228476 PMC8943384

[23] Gumber R Cohen JB Palmer MB Kobrin SM Vogl DT Wasserstein AG. A clone-directed approach may improve diagnosis and treatment of proliferative glomerulonephritis with monoclonal immunoglobulin deposits. Kidney Int. (2018) 94:199–205. 10.1016/j.kint.2018.02.020 29759418

[24] Hotta O Ieiri N Nagai M Tanaka A Harabuchi Y. Role of palatine tonsil and epipharyngeal lymphoid tissue in the development of glomerular active lesions (*Glomerular vasculitis*) in immunoglobulin a nephropathy. Int J Mol Sci. (2022) 23:727. 10.3390/ijms23020727 35054911 PMC8775943

